# Phytochemical, antioxidant and antimicrobial properties of *Litsea angulata* extracts

**DOI:** 10.12688/f1000research.16620.2

**Published:** 2019-03-20

**Authors:** Harlinda Kuspradini, Indah Wulandari, Agmi Sinta Putri, Sabeti Yulis Tiya, Irawan Wijaya Kusuma

**Affiliations:** 1 Forestry Faculty, Mulawarman University, Samarinda, East Kalimantan, 75123, Indonesia; 2 Lembaga Pendidikan Politeknik Malinau, North Kalimantan, Indonesia

**Keywords:** Litsea angulata, maceration, phytochemical, antioxidant, antimicrobial

## Abstract

**Background:** *Litsea angulata* is a plant species belonging to Lauraceae family that is distributed throughout Indonesia, Malaysia, and New Guinea. The seeds have been traditionally used by local people in Kalimantan, Indonesia for the treatment of boils; however, there is no information about the potency of its branch, bark and leaves yet. This study aimed to determine the antioxidant, antimicrobial activity as well as the phytochemical constituent of *Litsea angulata* branch, bark, and leaves.

**Methods:** Extraction was performed by successive maceration method using *n*-hexane, ethyl acetate, and ethanol solvent. Antioxidant activity was evaluated by DPPH radical scavenging assay. The antimicrobial activity using the 96 well-plate microdilution broth method against *Staphylococcus aureus* and *Streptococcus mutans*.

**Results:** Based on the phytochemical analysis, it showed that extract of *L. angulata* contains alkaloids, flavonoids, tannins, terpenoids, and coumarin. The results showed that all extracts of plant samples displayed the ability to inhibit DPPH free radical formation and all tested microorganisms.

**Conclusions:** *L. angulata* contains secondary metabolites such as alkaloids, flavonoids, tannins, terpenoids, carotenoids, and coumarin. The antioxidant activity on different plant extracts was a range as very strong to weak capacity. All extracts in this study could inhibit the growth of *S. aureus* and *S. mutans*.

## Introduction

Many plants species from the genus *Litsea* are a potential source of biologically active compounds and are used as traditional medicines such as antispasmodic, wound healing, relieving rheumatism and cold ^1, 2^. There is little information about the potency of *Litsea angulata* species *. L. angulata*, belonging to the Lauraceae family, which can be found in East Kalimantan, Indonesia, and to our knowledge, data are limited on its biological activities. The fruit seed of *L. angulata* has been reported to have antifertility properties. No previous studies report the antibacterial properties of *L. angulata* (bark, branch and leaves), while the related species such as *L. cubeba* leaf extract, *Litsea petiolata* Hook. f. leaves, and stem bark and leaf extracts of *Litsea glutinosa* (Lour.) C.B. Rob, presented antibacterial activity against *S.* aureus ^3– 5^. In addition essential oil of *Litsea cubeba* can also act as antibacterial against *S. mutans* ^6^. Therefore the present study aimed to assess the phytochemical constituents, antioxidant, and antimicrobial of different plant part of *L. angulata*.

## Methods

### Sample collection

The plant material was obtained from Education Forest Laboratory of Forestry Faculty, Mulawarman University, East Kalimantan, Indonesia (0° 25’10” – 0°25’24” Southern Latitude and, 117° 14’00”-117 14’14” East longitude). The soil texture, average air temperature and relativity humidity in this location were sandy loam, 25.4°C and 91.6%, respectively ^7, 8^. The plant samples were identified in Dendrology and Forest Ecology Laboratory of Forestry Faculty, Mulawarman University.

### Preparation of plant extracts

The plant material was obtained from Education Forest Laboratory of Forestry Faculty, Mulawarman University, East Kalimantan, Indonesia. Three different plant parts of *L. angulata* (bark, branch, and leaves) were separated, ground and extracted. The successive maceration extraction method was adopted from Sruthi and Indira ^9^, with the solvents modified. About 50 g of the air-dried powder of each plant material was extracted individually with one of the following solvents: *n*-hexane, ethyl acetate, and 96% ethanol. The extracts were filtered and concentrated under vacuum using a rotary evaporator until the solvent was completely evaporated. In total, nine different extracts were produced, with each solvent used to produce extract from each plant part (branch, *n*-hexane; branch, ethyl acetate; branch, ethanol; bark, *n*-hexane; bark, ethyl acetate; bark, ethanol; leaves, *n*-hexane; leaves, ethyl acetate, and leaves, ethanol extracts).

### Phytochemical screening

A total of 60 mg of each extract were dissolved individually in 1 ml solvent that used for extraction and the solutions were used to test for qualitative phytochemical tests. The tests were done according to the standard procedures described into literature by Kokate ^10^, Senthilmurugan ^11^, Harborne ^12^ to detect the following bioactive compounds: alkaloids, flavonoids, saponins, tannins, terpenoids, steroids, carotenoids, and coumarin.

### DPPH free radical scavenging assay

The DPPH assay was performed as described by Kuspradini *et al*. ^13^. Various concentrations of samples of each extract (12.5, 25, 50 and 100 ppm) in 96% ethanol were added with DPPH. After 20 minutes, the absorbance of the resulting solution and the blank were recorded. Ascorbic acid was used as a positive control. The absorbance was recorded spectrophotometrically at a wavelength of 517 nm. DPPH free radical scavenging activity was stated as % inhibition = (1 – Absorbance of sample/Absorbance of control) × 100. To inhibitory activity, half-maximal inhibitory concentration (IC50) values were calculated.

### Determination of antibacterial activity

The minimum inhibitory concentration (MIC) and the Minimum Bactericidal Concentration (MBC) of the samples were assessed against *Staphylococcus aureus* and *Streptococcus mutans* using the 96-well microdilution and solid medium, respectively. The bacterial concentration in the inoculum was standardized at 0.5 McFarland turbidity scale, equivalent to 10 ^8^ CFU ml ^–1^. The method of MIC was adopted from the method outlined by Mohsenipour and Hassanshahian, with modifications ^14^. A stock solution was prepared by dissolving 5 mg extracts in 1 ml of 40% ethanol. A total of 50 µl stock solution were serially diluted twofold in 40% ethanol to achieve the range of test concentrations (1250, 625, 312.5 and 156.25 ppm), which were added to wells of a 96-well microplate. Next, 100 µl sterile nutrient broth culture medium (NB) and 50 µl of the culture of the respective organism were added into each well. The inoculated microplates were incubated at 37°C for 24 h.

At 1 hour before the end of incubation, the bacterial growth was confirmed by adding 0.01% solution of 2,3,5-triphenyl tetrazolium chloride (TTC, Merck, Germany) (50 µl) and the plate was incubated for another hour. The viable bacterial cells reduced the yellow TTC to pink. The inhibition of growth was visually detected when the solution in the well remained clear after incubation with TTC. Positive controls (bacteria + NB + chloramphenicol), negative controls (bacteria and NB), vehicle controls (bacteria + NB + solvent), and media controls (NB) were included in each test. MBC was determined by inoculating the assay from the wells showing no microbial growth onto the surface of nutrient agar medium on the petri dish. The petri dishes were incubated for 24 h at 37°C and subjected to visual inspection. MBC was considered as the lowest concentration where there was no resumption of bacterial growth.

### Statistical analysis

All experiments were conducted three times. Regression analysis was used to calculate IC50 values of antioxidant. All statistical analyses used Microsoft Excel 2010 software.

## Results

### Phytochemical screening

The result of phytochemical screening showed that *L. angulata* contain alkaloid, flavonoid, tannin, terpenoid, carotenoid and coumarin ( Table 1). It can be shown that the ethanolic extract of the *L. angulata* showed more number of secondary metabolites when compared with other extracts. The extraction of various phytochemicals was seen to be more effectively done by ethanol (polar) than the ethyl acetate and n-hexane (semi polar and non polar) solvents.

**Table 1.** Secondary metabolites in *L. angulata* extracts.

Solvent	Part	Alkaloid	Flavonoid	Saponin	Tannin	Terpenoid	Steroid	Carotenoid	Coumarin
*n*-Hexane	Bark	+	+	-	-	+	-	-	-
	Branch	+	-	-	-	+	-	-	-
	Leaves	+	-	-	-	+	-	-	-
EtOAc	Bark	+	-	-	-	+	-	+	-
	Branch	+	-	-	-	+	-	-	-
	Leaves	+	-	-	-	+	-	+	-
EtOH	Bark	+	-	-	+	+	-	+	+
	Branch	+	-	-	+	+	-	+	+
	Leaves	-	+	-	+	+	-	+	+

### Antioxidant activity

All extracts could inhibit DPPH radical scavenging activity ( Table 2). The IC50 values with regards to different used solvents and plant parts were, in increasing order, as follows: bark, ethyl acetate; leaves-bark-branch, ethanol; branch, ethyl acetate; leaves, ethyl acetate; branch and leaves, n-hexane. Raw absorbance data from which IC50 values were calculated are shown in Dataset 1 ^15^.

**Table 2.** Half-maximal inhibitory concentration (IC50) values of *L. angulata* extracts on DPPH free radical.

No.	Solvent	Plant Part	IC50 (ppm)
1	*n*-hexane	Bark	76.12
		Branch	>100
		Leaves	>100
2	Ethyl acetate	Bark	2.41
		Branch	52.75
		Leaves	>100
3	Ethanol	Bark	14.69
		Branch	26.81
		Leaves	14.58

### Antimicrobial activity

All extracts could inhibit the growth of *S. mutans* and *S. aureus* and showed the MIC value at 156.25 ppm concentration ( Table 3). The MBC value could not detect in the range of 156.25–1250 ppm concentration. It is indicated that the MBC value in this study was higher than 1250 ppm. No pink color change can be seen in the negative control, vehicle control and media control. It confirms that the media (Nutrient Broth) and solvent did not affect the antimicrobial activity.

**Table 3.** Minimum inhibitory and bactericidal concentrations of the *L. angulata* extracts.

MIC (ppm)				
Solvent	Part	*S. mutans*	*S. aureus*	Control +
*n*-Hexane	Bark	156.25	156.25	100
	Branch	156.25	156.25	100
	Leaves	156.25	156.25	100
Ethyl acetate	Bark	156.25	156.25	100
	Branch	156.25	156.25	100
	Leaves	156.25	156.25	100
Ethanol	Bark	156.25	156.25	100
	Branch	156.25	156.25	100
	Leaves	156.25	156.25	100
**MBC (ppm)**				
*n*-Hexane	Bark	>1.250	>1.250	>1.250
	Branch	>1.250	>1.250	>1.250
	Leaves	>1.250	>1.250	>1.250
Ethyl acetate	Bark	>1.250	>1.250	>1.250
	Branch	>1.250	>1.250	>1.250
	Leaves	>1.250	>1.250	>1.250
Ethanol	Bark	>1.250	>1.250	>1.250
	Branch	>1.250	>1.250	>1.250
	Leaves	>1.250	>1.250	>1.250

Raw data associated with this study Data include the absorbance values obtained from the DPPH scavenging assay, and the resultant IC50 values generated. Click here for additional data file. Copyright: © 2019 Kuspradini H et al. 2019 Data associated with the article are available under the terms of the Creative Commons Zero "No rights reserved" data waiver (CC0 1.0 Public domain dedication).

## Discussion

Plant extracts have been reported to have numerous biological activities due to their phytochemical contents, which contribute significantly towards the antioxidant and antimicrobial activities such as flavonoids, tannins, and terpenoids ^16– 18^. The secondary metabolites extraction can be performed using solvent with different polarities, depending on the type of compound structure. Thus, it can be reported that polar solvent (ethanol) could extract more secondary metabolites such as tannin, carotenoid and coumarin than ethyl actetate and n-hexane solvent (semi-polar and non polar). It may be possibly be one of the reasons for a strong antioxidant activity shown by the ethanolic extracts of *L. angulata*. The solubility or insolubility of the active compound(s) in the solvent used for extraction can caused the differential effects on antioxidant and antibacterial ^19– 21^. The IC50 value was calculated using the regression linear equation, where y is 50 (percent inhibitory value) and x is amount of inhibitory concentration value in µg. Data analysis were shown in Dataset 1. According to Blois ^22^ sample which had an IC50 value more than 150 ppm was a weak antioxidant, 101–150 ppm was a medium antioxidant, 50–100 ppm indicated as a strong antioxidant, while lower than 50 ppm was a very strong antioxidant. Bark-ethyl acetate extract obtaining the lowest IC50 value (2.41 ppm) in comparison to another extracts, and it indicates that this extract has a strong antioxidant. Antimicrobials are considered as bactericidal if the MBC is not more than four times higher than the MIC, and MBC value is always equal or higher than MIC ^23^.

## Conclusions

*L. angulata* can be used as a source of natural antioxidant to prevent damage associated with DPPH free radicals and antibacterial to inhibit the growth of *S. mutans* and *S. aureus* bacteria. It may be due to the presence of its active compound such as alkaloids, flavonoids, tannins, terpenoids, carotenoids and coumarin.

## Data availability

The data referenced by this article are under copyright with the following copyright statement: Copyright: © 2019 Kuspradini H et al.

Data associated with the article are available under the terms of the Creative Commons Zero "No rights reserved" data waiver (CC0 1.0 Public domain dedication). 

Dataset 1. Raw data associated with this study. Data include the absorbance values obtained from the DPPH scavenging assay, and the resultant IC50 values generated. DOI: https://doi.org/10.5256/f1000research.16620.d223677 ^15^.
