# Supplementary material for: Phytochemical, antioxidant and antimicrobial properties of Litsea angulata extracts
Source: F1000Res. 2019 Mar 20;7:1839. Originally published 2018 Nov 22. [Version 2] doi: 10.12688/f1000research.16620.2 (PMC6357992; doi:10.12688/f1000research.16620.2)
Supplement: Peer review history

# Peer review: Reviewer response for version 2

**DOI:** 10.5256/f1000research.20243.r45953

**Published:** 2019-04-04

**Authors:** Sarifah Nurjanah

**Affiliations:** 1 Departement of Agricultural and Biosystem Engineering, Faculty of Agro-Industrial Technology, Padjadjaran University, Bandung, Indonesia

The manuscript has been updated according to the input given, so there is no more comment required.

I have read this submission. I believe that I have an appropriate level of expertise to confirm that it is of an acceptable scientific standard.

# Peer review: Reviewer response for version 2

**DOI:** 10.5256/f1000research.20243.r45954

**Published:** 2019-03-20

**Authors:** Mariateresa Cristani

**Affiliations:** 1 Department of Chemical, Biological, Pharmaceutical and Environmental Sciences, University of Messina, Messina, Italy

I have no other comments.

I have read this submission. I believe that I have an appropriate level of expertise to confirm that it is of an acceptable scientific standard.

# Peer review: Reviewer response for version 1

**DOI:** 10.5256/f1000research.18164.r43839

**Published:** 2019-01-31

**Authors:** Sarifah Nurjanah

**Affiliations:** 1 Departement of Agricultural and Biosystem Engineering, Faculty of Agro-Industrial Technology, Padjadjaran University, Bandung, Indonesia

This is an interesting manuscript containing new information on the potential of Litsea angulata. The paper is well written and provides valuable data. However, there are some suggestions to improve.

 1. Introduction:

- Not only include the reason for exploring the potential of Litsea angulate, but also why should it be seen from each part of the material (bark, branch and leaves), is there any previous research that each part of the plant has different active ingredients?
- It should also be written the reasons for using Staphylococcus aureus and Streptococcus mutans.

2. Results:

- An increase of IC50 value in antioxidant activity written in succession leaves, ethanol; bark, ethanol; ethanol branch; branch, ethyl acetate; bark, ethyl acetate; branch, n hexane; leaves and bark, n hexane; it should be bark, ethyl acetate; leaves, ethanol; bark, ethanol; ethanol branch; bark, ethyl acetate; branch, ethyl acetate; bark, n hexane; leaves ethyl acetate; branch and leaves n hexane.
- In the written method there are several controls used, namely positive control, negative control, vehicle control and control machines, but in the results only positive controls are written, what are the other controls?

3.  Discussion:

- Need clarification as to why ethanol can extract more active components such as tannin, carotenoid and coumarin compared to n hexane and ethyl acetate.
- Is there a relationship between antioxidant properties and antibacterial with component ingredients (phytochemical assessment results).

4. Conclusion:

- In conclusion section, only conclude about antioxidant activity and antibacterial activity. It should be mentioned also the conclusion of phytochemical assessment.

I have read this submission. I believe that I have an appropriate level of expertise to confirm that it is of an acceptable scientific standard, however I have significant reservations, as outlined above.

# Peer review: Reviewer response for version 1

**DOI:** 10.5256/f1000research.18164.r41035

**Published:** 2019-01-15

**Authors:** Morina Adfa

**Affiliations:** 1 Department of Chemistry, Faculty of Mathematics and Natural Sciences, University of Bengkulu, Bengkulu, Indonesia

Manuscripts entitled Phytochemicals, antioxidants and antimicrobial properties *Litsea* *angulata* extract has been written well. Revise in the keyword *Litsea* *angulata* in italic format, add your information where the species ( *Litsea* *angulata*) was determined. Please make sure the alkaloids contained in *n*-hexane extract because positive false in phytochemical tested are often reported. Authors wrote that all statistical analyses used Microsoft Excel 2010 software, but in the results and discussion, we cannot find the data with statistical analysis. The authors may add the data with statistical analysis or they can explain in the discussion because in the method they used the statistical analysis for data analysis. The authors may add the data with statistical analysis or they can explain in the discussion because in the method they used the statistical analysis for data analysis.

I have read this submission. I believe that I have an appropriate level of expertise to confirm that it is of an acceptable scientific standard.

# Peer review: Reviewer response for version 1

**DOI:** 10.5256/f1000research.18164.r41787

**Published:** 2019-01-07

**Authors:** Mariateresa Cristani

**Affiliations:** 1 Department of Chemical, Biological, Pharmaceutical and Environmental Sciences, University of Messina, Messina, Italy

I like the concept of this manuscript, and it could be interesting, but I think it needs some additional work. The only DPPH test is not enough to demonstrate the antioxidant activity and it is a easy test. Phytochemical screening is already known so it would be interesting at least HPLC analysis. The discussion could be a bit deeper explaining the benefits of extracts and comparing better which extracts is more useful and why. It would have been interesting to have information on the pedoclimatic characteristic of the place where the plant are harvested. The description of experimental part is for the most part good and clear. The written English needs to be better. The following reference which appeared recently in Natural Products Research, 2018 ^1^ should be added.

I have read this submission. I believe that I have an appropriate level of expertise to confirm that it is of an acceptable scientific standard, however I have significant reservations, as outlined above.

## References

[1] BoussahelSCacciolaFDahamnaSMondelloLSaijaACiminoFSpecialeACristaniM: Flavonoid profile, antioxidant and antiglycation properties of Retama sphaerocarpa fruits extracts. *Nat Prod Res*.2018;32(16) : 10.1080/14786419.2017.1356835 1911-1919 10.1080/14786419.2017.1356835 28738692
